# Determination of Larval Instars of *Ips hauseri* (Coleoptera: Curculionidae: Scolytinae), Using Kernel Density Estimation and Mixture Modeling of Head Capsule Widths

**DOI:** 10.3390/insects17070739

**Published:** 2026-07-20

**Authors:** Jipeng Jiao, Wei Zhang, Yunlong Liu, Yang Zhou, Mengli Zhang, Lili Ren, Adil Sattar

**Affiliations:** 1 College of Forestry and Landscape Architecture, Xinjiang Agricultural University, Urumqi 830052, China; jiaojipeng@xjau.edu.cn (J.J.); 18096993201@163.com (W.Z.); zy19834545375@163.com (Y.Z.); 17690990507@163.com (M.Z.); 2 Urumqi County Greening Management Station, Urumqi 830001, China; lul.zz@163.com; 3 Beijing Key Laboratory for Forest Pest Control, Beijing Forestry University, Beijing 100083, China; lily_ren@bjfu.edu.cn

**Keywords:** *Ips hauseri*, Scolytinae, larval instar, head capsule width, kernel density estimation, Gaussian mixture model, Brooks–Dyar rule, forest pest management

## Abstract

*Ips hauseri* (Coleoptera: Curculionidae: Scolytinae) is a destructive stem pest of *Picea schrenkiana* var. *tianshanica*, and determining its larval instars is essential for monitoring population development and timing control. Healthy spruce logs were deployed to attract ovipositing adults, and 553 larvae were collected through sequential destructive sampling of previously unsampled logs from June to July 2025. Head capsule widths were measured and analysed using kernel density estimation (KDE) and a Gaussian mixture model (GMM), and the classification was biologically validated against the theoretical growth expectation of the Brooks–Dyar rule, together with the Crosby growth-ratio test and linear regression. Head capsule widths showed a clear trimodal distribution corresponding to three instars, with mean widths of 515.82 ± 21.50, 676.01 ± 27.22, and 885.96 ± 38.27 μm for instars I–III. The mean Brooks–Dyar ratio was 1.311, and the natural logarithm of head capsule width was almost perfectly linear with instar number (R^2^ > 0.999; *p* < 0.001). This study provides the first quantitative evidence that *I. hauseri* has three larval instars, offering a basis for studying its developmental biology and improving population forecasting and precise management.

## Simple Summary

*Ips hauseri* is one of the most damaging insects attacking *Picea schrenkiana* var. *tianshanica* forests in the Tianshan Mountains, where it bores into the trunk and can kill trees over large areas. Effective management of this pest depends on understanding how its larvae develop, yet the number of larval growth stages (instars) has not been previously established. In this study, we reared the beetle on healthy spruce logs, collected 553 larvae, and measured the width of their head capsules—a structure that enlarges in fixed steps at each molt and is widely used to distinguish larval stages. Using several complementary statistical methods, we found that the larvae pass through three instars. These results clarify the life cycle of the species and provide a basis for predicting its development and for timing control measures more precisely.

## 1. Introduction

*Picea schrenkiana* var. *tianshanica* is the edificator species of the Tianshan Mountain forest ecosystem and plays an irreplaceable role in water conservation and the maintenance of alpine biodiversity. In recent years, influenced by climate warming and the increasing frequency of extreme drought events, *P. schrenkiana* forests have experienced large-scale infestations by bark beetles, resulting in severe tree decline and widespread mortality. Among these, *Ips hauseri* Reitter, 1894 (Coleoptera: Curculionidae: Scolytinae), a native and dominant stem pest, has emerged as a critical biotic agent threatening the health of these forests. By tunneling into the phloem and xylem, *I. hauseri* directly disrupts the vascular transport system of host trees, leading to a rapid deterioration of tree vigor and ultimately contributing to extensive forest dieback.

Accurate assessment of population developmental dynamics is fundamental to the implementation of precise pest management, and the classification of larval instars constitutes a critical step in resolving the developmental trajectory of insect pests. In entomological research, the width of the larval head capsule is widely regarded as the most reliable morphometric indicator for instar discrimination because it increases in a stepwise manner during molting and remains unaffected by post-feeding contraction [1,2,3]. Traditional methods of instar classification have relied heavily on the visual interpretation of frequency distribution histograms of head capsule widths, an approach that is inherently subjective and prone to significant error, particularly when sample sizes are large and distributions overlap considerably between successive instars [4,5].

With the increasing application of advanced statistical techniques, objective classification methods based on Kernel Density Estimation (KDE) and Gaussian Mixture Models (GMM) have become the prevailing standard. By fitting multimodal distributions to head capsule width data and employing the Expectation-Maximization (EM) algorithm, these approaches automatically estimate the mean, variance, and mixing proportion for each instar, thereby effectively circumventing the arbitrariness associated with manual classification [6,7,8,9].

Furthermore, the biological validity of instar classification must be verified against established growth patterns. As a theoretical principle of insect growth, the Brooks–Dyar rule posits that the head capsule width of insect larvae progresses geometrically between successive instars, resulting in a linear relationship between the natural logarithm of mean head capsule width and the instar number. In the present study, this rule served as a biological validation criterion for evaluating instar groupings derived from empirical measurements and statistical models, rather than as a source of data.

To date, research on *I. hauseri* has primarily focused on its infestation characteristics, spatial distribution patterns, and adult biology, whereas systematic and quantitative investigations into the classification of its larval instars remain lacking. Given that the larval stage constitutes the critical period for feeding damage and nutrient accumulation, elucidating the instar structure is essential for understanding population developmental phenology, forecasting emergence timing, and assessing infestation severity. Accordingly, the present study conducted extensive dissections of colonized host material and employed a combination of statistical approaches—including Kernel Density Estimation (KDE) and Gaussian Mixture Modeling (GMM)—to delineate the larval instars of *I. hauseri*. The biological validity of the classification scheme was further corroborated using the Brooks–Dyar rule. The overarching aim of this work is to provide a foundational basis for research on the developmental biology of this pest and to support the development of accurate monitoring and forecasting programs.

## 2. Materials and Methods

### 2.1. Larval Collection and Species Identification

On 16 June 2025, twenty-six uninfested and healthy log sections of *Picea schrenkiana* var. *tianshanica* were deployed in a forest stand severely infested by *I. hauseri* within the Urumqi Banfanggou Branch of the Eastern Tianshan State-Owned Forest Administration, Xinjiang (87°14′09″ E, 43°25′24″ N). The log sections were cut from the trunks of several freshly felled, healthy and uninfested trees. Each trunk was cross-cut into consecutive 1 m sections from the base upward, and all sections from the butt and middle portions of the bole were retained, that is, from the stump up to the height at which the over-bark diameter decreased to approximately 10 cm; the thin upper bole and the crown were discarded. The over-bark diameter of the retained sections therefore ranged from 10 to 20 cm, and each section was 1 m in length. The log sections were intended to attract flying adult *I. hauseri* to colonize and oviposit naturally. Following a four-day exposure period, once a sufficient density of entrance holes had been observed, all log sections were transported to an outdoor enclosure at a lower elevation. The cut ends of each log were sealed with paraffin wax to minimize desiccation and maintain near-natural moisture conditions. Subsequently, all log sections were enclosed individually within fine-mesh netting.

Because several scolytine species, notably *Pityogenes spessivtsevi*, can coinfest *P. schrenkiana* var. *tianshanica* in the study area, the identity of the colonizing beetles was confirmed before larval collection. Adults extracted from the entrance holes and nuptial chambers were identified as *I. hauseri* following the diagnostic characters described by Wang et al. [10]: the elytral declivity bears four spines on each side, of which the second and third arise from a common base and the four spines are equidistantly spaced, and the frons bears a prominent tubercle slightly below its centre that projects among the frontal granules; these characters, together with its larger body size, clearly separated *I. hauseri* from the much smaller *P. spessivtsevi*. Species identity was further corroborated by the maternal gallery system—longitudinal egg galleries radiating from a central nuptial chamber, the stellate polygamous pattern typical of *Ips*—and by the round entrance holes marked externally by reddish-brown boring frass. Because the log sections were enclosed in fine-mesh netting after transfer, no further colonization could occur, and their bark surface was inspected at each sampling occasion to confirm the absence of new entrance holes, ensuring that all dissected larvae originated from the verified *I. hauseri* colonization.

Commencing from the time of adult beetle entry into the log sections, one previously un-sampled infested log was randomly selected every four days, and five bark samples (each measuring 20 cm × 30 cm) were excised and carefully dissected to retrieve larvae of *I. hauseri* from the subcortical tissues. This sequential destructive sampling procedure was conducted at four-day intervals until all larvae within the logs had completed development and pupated. All collected larval specimens were preserved in 75% ethanol, transported to the laboratory, and stored at 4 °C for subsequent morphometric analysis.

### 2.2. Head Capsule Width Measurement

The head capsule widths of *I. hauseri* larvae were measured using a stereomicroscope (XTL-BM-18TD; Shanghai BM Optical Instrument Manufacture Co., Ltd., Shanghai, China) equipped with a digital imaging system, which was employed to capture photographs of each specimen. The maximum width of each head capsule was subsequently determined from the digital images using Capture 2.1 image analysis software (Shanghai BM Optical Instrument Manufacture Co., Ltd., Shanghai, China), with a measurement precision of 0.01 mm (10 μm).

### 2.3. Larval Instar Determination

#### 2.3.1. Exploration of Data Distribution Characteristics

The distributional characteristics of the data were explored using a combination of Kernel Density Estimation (KDE) and frequency distribution analysis. Nonparametric density estimation was performed using the density() function in R (version 4.5.2; R Foundation for Statistical Computing, Vienna, Austria) with a Gaussian kernel. The smoothing bandwidth was selected using Silverman’s rule-of-thumb estimator [11], the default method (bw.nrd0) of the density() function, given by h = 0.9 × min(SD, IQR/1.349) × n^−1/5^, where SD is the sample standard deviation, IQR the interquartile range, and n the sample size; for the present dataset (n = 553) this yielded h = 37.63 μm. Frequency distributions were constructed by generating histograms with varying numbers of bins (20, 30, 40, and 50 bins). Following visual comparison, a bin number of 30 was ultimately selected for the frequency distribution analysis, as this configuration optimally resolved the multimodal characteristics of the data.

#### 2.3.2. Gaussian Mixture Model Fitting and Instar Assignment

Based on finite mixture model theory, a Gaussian Mixture Model (GMM) was constructed for instar identification. The model assumed that the head capsule width data originated from a weighted mixture of k normal distributions. Parameter estimation was implemented using the normalmixEM() function from the R package mixtools (version 2.0.0), which employs the Expectation-Maximization (EM) algorithm for model fitting. The number of mixture components, k, was specified according to the number of distinct peaks observed in the preliminary frequency distribution analysis. Model parameters were estimated via the EM algorithm, and instar assignment was conducted based on the principle of maximum posterior probability, whereby each individual was allocated to the instar category exhibiting the highest posterior probability.

The component means and standard deviations estimated by the GMM via the EM algorithm were used as the instar-specific parameter estimates, and the corresponding instar-identification thresholds were defined as the estimated component mean ± 2 SD. In addition, a nonlinear least-squares (NLLS) procedure was used to fit a normal density curve to each instar subset, providing the fitted curves overlaid on the head capsule width histogram for visualization.

#### 2.3.3. Validation of Instar Classification Using the Brooks–Dyar Rule

To validate the biological plausibility of the instar classification scheme, the Brooks–Dyar rule was applied as a theoretical principle and biological validation criterion to the observed and GMM-estimated instar means; it was not used to generate or estimate head capsule width data. Specifically, the Brooks–Dyar ratio was calculated according to Equation (1), while Crosby’s growth rule—which detects overlooked instars by comparing the proportional difference between successive Brooks–Dyar ratios against an approximate 10% threshold—was applied according to Equation (2), where X_n_ represents the mean head capsule width of the n-th instar, and X_n−1_ represents the mean head capsule width of the (n − 1)-th instar. Linear regression analysis was performed separately on the GMM-estimated mean head capsule widths and the observed mean head capsule widths. A linear model relating the natural logarithm of head capsule width to instar number was established, and the goodness-of-fit was evaluated using the coefficient of determination (R^2^). The regression analysis also served to verify conformity to the geometric growth principle described by the Brooks–Dyar rule, thereby ensuring that the instar classification adheres to biologically meaningful patterns of insect development.

BD = X_n_/X_n−1_ (1)

CR = (BD_n_ − BD_n−1_)/BD_n−1_ (2)

## 3. Results

### 3.1. Observed Characteristics of Head Capsule Widths by Instar

**Figure 1:**
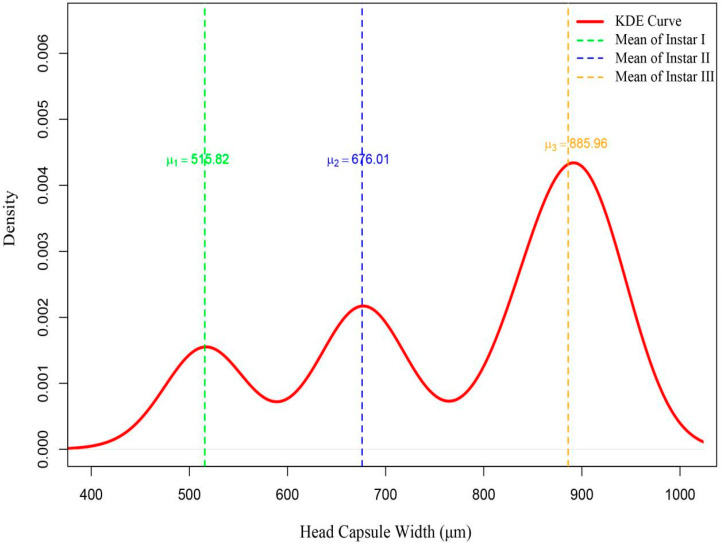
Kernel Density Estimation of Head Capsule Width and Distribution of Instar Means.

A total of 553 larval specimens of *I. hauseri* were measured for head capsule width, encompassing the complete developmental cycle of the species. Frequency distribution analysis of head capsule widths revealed a distinct trimodal distribution pattern. Kernel Density Estimation (KDE) with a bandwidth of 37.63 μm clearly resolved three well-separated distribution peaks (Figure 1), providing preliminary evidence for the existence of three larval instars. On this basis, the 553 larvae were assigned to three instars using the Gaussian mixture model detailed in Section 3.2, and descriptive statistics of the resulting groups were then calculated (Table 1). The results indicated that Instar I (n = 91) exhibited a mean head capsule width of 515.82 ± 21.50 μm (range: 430–580 μm). Instar II (n = 138) had a mean width of 676.01 ± 27.22 μm (range: 600–740 μm), representing a marked increase relative to Instar I. Instar III (n = 324) attained the largest dimensions, with a mean width of 885.96 ± 38.27 μm (range: 770–980 μm). The Brooks–Dyar ratios calculated from the observed means were 1.311 for Instar II/Instar I and 1.311 for Instar III/Instar II, yielding an average growth ratio of 1.311, a pattern consistent with the geometric progression of insect growth. Applying Crosby’s growth rule, the two successive Brooks–Dyar ratios differed by less than 0.1%, far below the ~10% threshold, indicating that no larval instar had been overlooked. Coefficients of variation within each instar were consistently low (4.03–4.32%), reflecting a high degree of homogeneity in individual body size within a given instar. This classification is robustly supported by both kernel density estimation and growth ratio analyses, thereby establishing a solid empirical foundation for subsequent model-based validation.

### 3.2. Gaussian Mixture Model-Based Instar Classification

**Figure 2:**
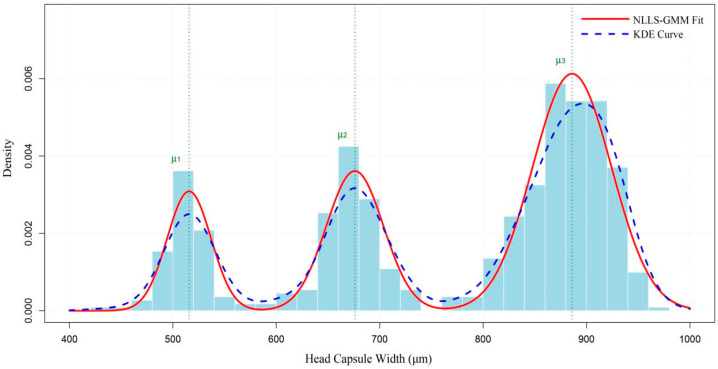
Head Capsule Width Distribution and NLLS-GMM Fit (Histogram, KDE, and GMM Comparison).

To overcome the subjectivity inherent in traditional morphometric approaches to instar classification, a Gaussian Mixture Model (GMM) was employed to objectively identify larval instars from the head capsule width data. Based on the three distinct peaks revealed by Kernel Density Estimation, the number of mixture components was set to k = 3. The goodness-of-fit of the GMM to the observed data is illustrated in Figure 2. The fitted model curves closely matched the observed data histogram, with the three Gaussian components corresponding clearly to the three instar peaks and exhibiting minimal overlap among adjacent components. The 553 individual measurements were assigned to three normally distributed components via the GMM.

The GMM-estimated parameters are summarized in Table 2. Instar I exhibited a GMM-estimated mean head capsule width of 515.69 ± 21.22 μm (CV = 4.11%), Instar II had a GMM-estimated mean of 676.02 ± 27.64 μm (CV = 4.09%), and Instar III showed a GMM-estimated mean of 886.02 ± 38.13 μm (CV = 4.30%). The GMM-estimated size ranges for each instar were well defined: Instar I (<558 μm), Instar II (621–731 μm), and Instar III (>810 μm), with negligible overlap between successive instars. Differences between the GMM-estimated and observed means were less than 0.03% for each instar, and discrepancies in standard deviations were within 2%, indicating a high degree of model fit. The Brooks–Dyar ratios calculated from the GMM-estimated means were uniformly 1.311, consistent with those obtained from the observed data, further corroborating the reliability of the model.

Kernel Density Estimation provided independent support for the three-instar classification scheme. The KDE curve, fitted with a bandwidth of 37.63 μm, resolved three well-separated distribution peaks whose locations corresponded closely to the mean values identified by the GMM (deviation < 0.25%). The standardized bandwidth, expressed as a z-score, was 0.2545. The convergent results of the Gaussian Mixture Model and Kernel Density Estimation robustly support the conclusion that the larval development of *I. hauseri* comprises three distinct instars. The GMM not only furnishes an objective framework for instar classification but also provides precise parameter estimates for head capsule width in each instar, thereby establishing a reliable statistical foundation for subsequent biological investigations.

### 3.3. Regression-Based Validation of Instar Classification

**Figure 3:**
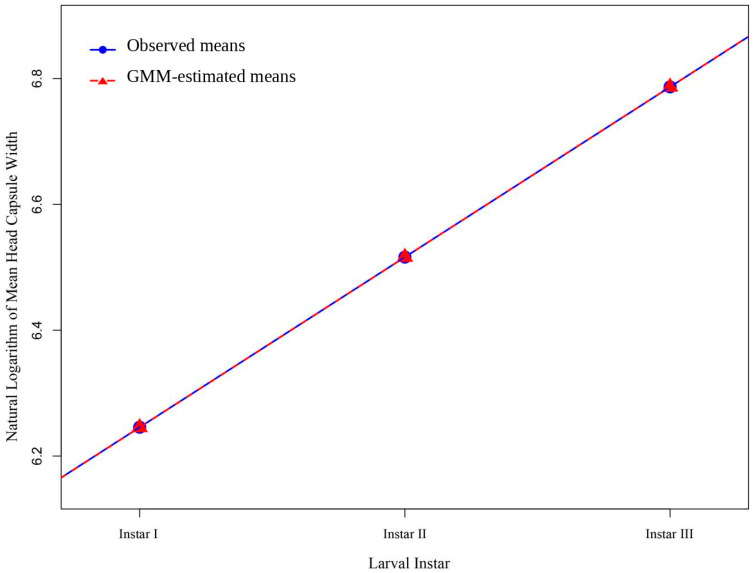
Comparison of GMM-Estimated and Observed Mean Head Capsule Widths across Larval Instars.

To validate the biological plausibility of the instar classification from the perspective of growth patterns, linear regression analysis was performed relating instar number to the natural logarithm of head capsule width. Based on the geometric growth principle articulated by the Brooks–Dyar rule, regression models were constructed separately for the observed means and the GMM-estimated means. The results of the regression analysis indicated that the coefficient of determination (R^2^) was 0.999 for the observed means and greater than 0.999 for the GMM-estimated means, demonstrating that instar number accounted for more than 99.9% of the variance in head capsule width (*p* < 0.001). The regression equation for the observed means was ln(head capsule width) = 5.9745 + 0.2704 × instar, whereas that for the GMM-estimated means was ln(head capsule width) = 5.9749 + 0.2706 × instar. The two regression lines were virtually coincident, with a difference in slopes of only 0.0002. The scatter plot and fitted regression curves in Figure 3 graphically illustrate the strict linear relationship between instar and head capsule width, with the mean log-transformed head capsule widths of each instar clustered tightly about the regression line. The GMM-estimated and observed means exhibited a high degree of congruence in the regression analysis. The regression-implied Brooks–Dyar ratio derived from the slope was 1.311, which precisely matched the average ratio computed directly from the data, thereby mathematically corroborating the geometric progression characteristic of larval growth. It should be emphasised that this regularity resides in the instar means: although the mean Brooks–Dyar ratio is nearly constant, individual head capsule widths within each instar still varied appreciably (CV = 4.0–4.3%; SD = 21–38 μm), so the raw measurements retain natural biological variance and were neither smoothed nor back-generated from the model. Such close adherence to Dyar’s rule, together with a Crosby growth-rule difference of <0.1% between successive ratios (well within the ~10% criterion), indicates a complete instar series with no overlooked stage rather than an artefact of data processing. The regression validation results confirm that the increase in head capsule width of *I. hauseri* larvae adheres rigorously to the Brooks–Dyar rule, and that the three-instar classification scheme is substantiated on both statistical and developmental biological grounds.

## 4. Discussion

In the present study, the head capsule widths of 553 larvae were measured to determine the number of larval instars of *I. hauseri*. This three-instar pattern is consistent with the condition most frequently reported within the tribe Ipini. A comprehensive review of 23 bark beetle species by Lekander [12] indicates that the number of larval instars within the Scolytinae varies among species, ranging from two to five, with three instars being the most prevalent condition, particularly within the tribe Ipini. For instance, Lekander reported three larval instars for *Pityogenes chalcographus* (L.), *Pityogenes quadridens*, *Ips sexdentatus*, *Ips typographus* (L.), *Orthotomicus proximus*, and *Orthotomicus suturalis*, while Wilkinson documented three instars for *Ips grandicollis* and *Ips avulsus* [13]. The findings of the present study add a further example to this established pattern. Moreover, our results are consistent with multiple investigations identifying head capsule width as a reliable and effective morphometric parameter for discriminating larval instars in scolytid beetles [5,9,14,15,16,17].

The finding of three instars also invites brief consideration of why this number might be adaptive for *I. hauseri*. In bark beetles, instar number is generally a conserved trait, whereas developmental rate, adult body size, and voltinism respond more plastically to temperature and host quality. *I. hauseri* clearly exemplifies such plasticity in voltinism: historically univoltine in the Tianshan, it now completes two generations per year in the warming eastern part of the range, and its voltinism shifts elevationally from one generation per year at high elevations to two at lower, warmer elevations [18]. A fixed programme of three instars is plausibly adaptive in this context, since minimizing the number of molts shortens the larval period and allows the subcortical brood to exploit the ephemeral phloem of a dying host and to complete development within a short montane growing season—an advantage that is accentuated under the compressed bivoltine schedule now observed. Adding a generation while retaining three instars, rather than altering instar number, parallels the closely related *I. typographus*, whose larvae consistently pass through three instars [12] even though its voltinism ranges from uni- to multivoltine across its distribution. By contrast, some other bark beetles, such as *Dendroctonus*, can vary between three and four instars under different conditions, indicating that developmental plasticity in instar number differs among genera. Whether the instar number of *I. hauseri* itself varies geographically or thermally nonetheless remains untested, since the present sample derives from a single low-elevation, bivoltine population; comparative study of high-elevation, univoltine populations would be a valuable next step for establishing whether three instars is invariant across the species’ ecological range.

With respect to the methodology of instar determination, the combined application of Kernel Density Estimation (KDE) and Gaussian Mixture Modeling (GMM) in the present study provides a more objective and quantitative classification framework compared to earlier investigations that relied primarily on visual inspection of frequency distribution histograms of head capsule widths [5,19]. KDE effectively resolved the multimodal structure of the data, whereas GMM, through the Expectation-Maximization (EM) algorithm, enabled precise estimation of instar-specific parameters, including means, standard deviations, and GMM-estimated size ranges [7,9,14]. The high degree of congruence between GMM-estimated and observed values—evident in both mean estimates and standard deviations—attests to the robustness and reliability of the statistical model. Furthermore, the coefficients of variation (CV) within each instar were consistently low (<4.32%), indicating a high degree of morphometric homogeneity among individuals of the same instar and thereby providing indirect corroboration of the accuracy of the model-based classification.

The most compelling biological validation of the present findings comes from their conformity to the theoretical growth principle embodied in the Brooks–Dyar rule. The computed Brooks–Dyar ratios between successive instars were consistently 1.311, a value that aligns closely with the ranges reported in the literature for diverse insect taxa, including *Dendroctonus valens* (1.33) [20], *Eucryptorrhynchus brandti* (1.1–1.5) [21], *Heilipus lauri* (1.42) [22], and *Chironomus sancticaroli* (1.67) [23]. More critically, the exceptional linear relationship observed between the natural logarithm of head capsule width and instar number (R^2^ > 0.999) provides robust evidence that the growth of the larval head capsule adheres rigorously to a geometric progression, thereby corroborating the biological validity of the three-instar classification from a developmental perspective. This strict linear growth pattern has been similarly well documented in other insect species, including *Dastarcus helophoroides* (Coleoptera: Bothrideridae) [9], *Dendrolimus pini* (Lepidoptera: Lasiocampidae) [24], *Acanthoscelides macrophthalmus* (Coleoptera: Chrysomelidae) [25], *Conotrachelus perseae* (Coleoptera: Curculionidae) [26], and *Anagotus fairburni* (Coleoptera: Curculionidae) [27].

Although the number of instars in *I. hauseri* conforms to the general pattern observed within its tribe Ipini, it is important to acknowledge that inter-specific variation in instar number does exist across the Scolytinae. For instance, species within the tribe Hylastini, such as *Hylastes brunneus* Erichson and *Hylurgops palliatus*, possess four larval instars. Within the tribe Hylurgini, members of the genus *Dendroctonus* (e.g., *D. ponderosae* and *D. frontalis*) predominantly exhibit four instars, whereas *D. micans* undergoes five instars [14]. Conversely, certain species, such as *Pityophthorus confertus* Swaine of the tribe Corthylini, are reported to have only two instars [5]. Such interspecific variation likely reflects adaptive evolution to distinct ecological niches, host characteristics, and prevailing environmental pressures. In the case of *I. hauseri*, retaining three instars is consistent with this broader pattern of niche-related adaptation and, as discussed above, may reflect a life-history strategy suited to the short montane growing season of the Tianshan Mountains.

It should be noted that the present study relied primarily on morphometric data for instar classification. Although the application of statistical models substantially reduces subjective bias, a certain probability of misclassification persists in regions where the head capsule width distributions of adjacent instars overlap. Furthermore, larval development may be influenced by a variety of factors, including environmental conditions, intraspecific competition, sex, and pathogen infection, any of which could induce atypical growth in individual specimens and thereby complicate accurate instar assignment. Future investigations could incorporate microscopic morphological characters that differ among instars—such as features of the mouthparts—as auxiliary criteria to further refine the classification scheme.

In summary, this study has integrated morphometric measurements, Kernel Density Estimation (KDE), Gaussian Mixture Modeling (GMM), and growth pattern analysis to provide convergent and unequivocal evidence that the larval stage of *I. hauseri* comprises three distinct instars. This finding not only aligns with the general pattern observed among phylogenetically related species within the tribe Ipini, but the calculated growth ratios also conform to the prevailing patterns documented for scolytid beetles, thereby establishing a foundational framework for understanding the biology of this species.

## 5. Conclusions

This study provides the first quantitative determination of the larval instar structure of *I. hauseri*. The observed data demonstrate that *I. hauseri* undergoes three distinct larval instars during its development, with mean head capsule widths of approximately 516, 676, and 886 μm. This conclusion was supported by kernel density estimation, Gaussian mixture modeling, nonlinear least-squares fitting, the Brooks–Dyar rule, and linear regression. Head capsule growth follows a strict geometric progression (growth ratio of approximately 1.31; coefficient of determination greater than 0.999), consistent with the pattern that prevails in the tribe Ipini. The instar criteria established here provide a practical tool for staging field populations and a foundation for further research on the developmental biology, phenology, and precise management of this important forest pest.

## Figures and Tables

**Table 1:** Statistics of Observed Head Capsule Widths for Each Instar.

Instar	n	Mean ± SD (μm)	Range (μm)	CV (%)	Brooks–Dyar Ratio	Crosby’s Ratio
1	91	515.82 ± 21.50	430–580	4.17	–	–
2	138	676.01 ± 27.22	600–740	4.03	1.311	–
3	324	885.96 ± 38.27	770–980	4.32	1.311	0.000

Note: Mean ± SD: mean ± standard deviation; CV: coefficient of variation.

**Table 2:** GMM-Estimated Parameters and Instar Identification Thresholds.

Instar	n	Mean ± SD (μm)	GMM-Estimated Range (μ ± 2σ)	CV (%)	Brooks–Dyar Ratio
1	91	515.69 ± 21.22	<558 μm	4.11	–
2	138	676.02 ± 27.64	621–731 μm	4.09	1.311
3	324	886.02 ± 38.13	>810 μm	4.30	1.311

Note: Mean ± SD: mean ± standard deviation; CV: coefficient of variation; GMM-estimated range: range defined using the GMM-estimated mean and standard deviation (μ ± 2σ).

## Data Availability

The original contributions presented in this study are included in the article. Further inquiries can be directed to the corresponding author(s).
